# Supplementary material for: 2,4-Thiazolidinedione in Well-Fed Lactating Dairy Goats: II. Response to Intra-Mammary Infection
Source: Vet Sci. 2019 Jun 5;6(2):52. doi: 10.3390/vetsci6020052 (PMC6632143; doi:10.3390/vetsci6020052)
Supplement: Supplementary file 1 [file vetsci-06-00052-s001.zip › Table S1.docx]

**Table S1.** Intra- and inter-assay variation of the several parameters measured in plasma. See [[1](#_ENREF_1)] for parameters not reported here.

|  | CV between-assay | CV within-assay | sensitivity |
| --- | --- | --- | --- |
| Haptoglobin | 13.5 | 6.8 | 0.01 g/L |
| Total bilirubin | 6.7 | 2.7 | 0.3 mcmol/L |
| Total cholesterol | 2.1 | 1.7 | 0.2 mmol/L |
| Paraoxonase | 6.8 | 2.2 | 10 U/mL |
| FRAP | 7.7 | 2.8 | 20 mcmol/L |
| ROM | 12.5 | 1.6 | 3 mg H_2_O_2_/100 mL |
