# Supplementary material for: 2,4-Thiazolidinedione in Well-Fed Lactating Dairy Goats: II. Response to Intra-Mammary Infection
Source: Vet Sci. 2019 Jun 5;6(2):52. doi: 10.3390/vetsci6020052 (PMC6632143; doi:10.3390/vetsci6020052)
Supplement: Supplementary file 1 [file vetsci-06-00052-s001.zip › Table S2.docx]

**Table S2**. Primer-pairs newly designed for the experiment.

| **Gene** | **Full name** | **NCBI acc. #** | **Start** | **Primer** | **Am Size** | **Am Tm** |
| --- | --- | --- | --- | --- | --- | --- |
| *APOE* | apolipoprotein E | XM_018062563.1 | F-347 | GGCCGCCTCTGGGATTAC | 98 | 85 |
| *.* | . | . | R-444 | AGCACCGTCAGTTCCTGAATG |  |  |
| *ARG1* | Arginase 1 | NM_001285705.1 | F-383 | TGTGTCATTTGGGTGGATGCT | 120 | 79 |
| *.* | . | . | R-502 | CTCGGGCATCTTTTCCTTTAGTT |  |  |
| *CD14* | Cluster of differentiation 14 | NM_001285583.1 | F-141 | TAGCGCCGTTCAGTGTATGG | 102 | 85 |
| *.* | . | . | R-242 | TACTGCTTCGGGTCGGTGTT |  |  |
| *CD36* | CD36 molecule | NM_001285578.1 | F-1243 | TTTGGCTTAATGAGACTGGTACCA | 90 | 79 |
| *.* | . | . | R-1332 | CCAGGCCAAGGAGGTTTATTT |  |  |
| *CPT1A* | carnitine palmitoyltransferase 1A. | XM_018043311.1 | F-274 | CATCAGATTCAAGAATGGCATCA | 100 | 82 |
| *.* |  | . | R-373 | CTTGGCGTACATGGTCGACAT |  |  |
| *FABP4* | adipocyte fatty acid-binding protein. | EF105407.1 | F-68 | AAGTGGGTGTGGGCTTTGC | 107 | 79 |
| *.* |  | . | R-174 | AAAGGTGCTTTCTGATTTAATGTTGA |  |  |
| *HP* | haptoglobin | XM_005692202.3 | F-522 | GAATGTGAGGCAGTGTGTGGAA | 143 | 83 |
| *.* | . | . | R-664 | AGTGTGGCTCCCGAGATGAG |  |  |
| *IL10* | interleukin 10 | XM_005690416.2 | F-731 | CGGCGCTGTCATCGTTTT | 100 | 81 |
| *.* | . | . | R-830 | CTTTGTAGACACCCCTCTCTTGGA |  |  |
| *IL4* | interleukin 4 | NM_001285681.1 | F-229 | CTGCCCCAAAGAATGCAACT | 90 | 80 |
| *.* | . | . | R-318 | TGTTCAAGCACATGTGGTTCCT |  |  |
| *MRC1* | mannose receptor C-type 1. | XM_005687981.3 | F-3299 | GTCTTTCTTACGAAGATGCTGACTGT | 113 | 78 |
| *.* |  | . | R-3411 | TGTCTGACATATATAACCTCGCTTACTG |  |  |
| *NFKB1* | nuclear factor of kappa light polypeptide gene enhancer in B-cells 1 | XM_005681365.2 | F-528 | GGTGGTCGGCTTTGCAAA | 91 | 78 |
| *.* |  | . | R-618 | TACACAGGCGTCTGTCATTCG |  |  |
| *NOS2* | nitric oxide synthase 2. | XM_013971952.1 | F-492 | CTTCAAAGAGGCAAAAATAGAGGAA | 120 | 80 |
| *.* |  | . | R-611 | GCCTGCTTGGTGGCAAAG |  |  |
| *PDK4* | pyruvate dehydrogenase kinase 4. | XM_005678949.3 | F-1041 | CTGATGAACCAGCACATCCTTATATT | 92 | 79 |
| *.* |  | . | R-1132 | GCCGCCACATCACAGTTTG |  |  |
| *SUMO1* | small ubiquitin-like modifier 1. | XM_018060803.1 | F-202 | GCCTTACTCTGCAGGAAGCAA | 100 | 78 |
| *.* |  | . | R-301 | TCACTGCTATCCTGTCCAATGACT |  |  |
| TGFB1 | transforming growth factor beta 1. | NM_001314142.1 | F-850 | TACTGCTTCAGCTCCACAGAAAAG | 100 | 81 |
| . |  | . | R-949 | ACCCCTTGGGTTCGTGAATC |  |  |
