# Supplementary material for: 2,4-Thiazolidinedione in Well-Fed Lactating Dairy Goats: II. Response to Intra-Mammary Infection
Source: Vet Sci. 2019 Jun 5;6(2):52. doi: 10.3390/vetsci6020052 (PMC6632143; doi:10.3390/vetsci6020052)
Supplement: Supplementary file 1 [file vetsci-06-00052-s001.zip › Table S3.docx]

**Table S3**. Sequencing results of amplicons from primer-pairs from Table S2

| ***Gene*** | ***E-value*** | ***Primer-Blast*** | ***Sanger Sequencing*** |
| --- | --- | --- | --- |
| CD14 | 1.00E-20 | PREDICTED: Capra hircus CD14 molecule (CD14), mRNA | CGTCGTGGCGGCGGCACAGCCTGGACCAGTTTCTCAAGGGAGCCAACACCGACCCGAAGCAGTA |
| IL10 | 3.00E-21 | PREDICTED: Capra hircus interleukin 10 (IL10), mRNA | GCAGAGCAGCGGTGGAGCAGGTGAAGAGAGTCTTCAATATGCTCCAAGAGAGGGGTGTCTACAAAGA |
| TGFB1 | 8.00E-23 | PREDICTED: Capra hircus transforming growth factor beta 1 (TGFB1), transcript variant X1, mRNA | CAAGGTCGTCGCTCTACATTGACTTCCGGAAGGACCTGGGCTGGAAGTGGATTCACGAACCCAAGGGGTA |
| NOS2 | 1.00E-27 | PREDICTED: Capra hircus nitric oxide synthase 2 (NOS2), mRNA | GCGGGAGCGGTACAAGGAGATAGAAACAACAGGAACCTACCAGCTGACGGGAGATGAGCTCATCTTTGCCACCAAGCAGGCA |
| CD36 | 6.00E-12 | PREDICTED: Capra hircus CD36 molecule (CD36), transcript variant X5, mRNA | TAAAGCGGATGTTCAGAAGCAAGTGACGGGGAAAATAAACCTCCTTGGCCTGGA |
| IL4 | 1.00E-14 | Capra hircus interleukin 4 (IL4), mRNA | GAGGAGAGGCTGGATTGAGCTTAGGCGTATCTACAGGAACCACATGTGCTTGAACAA |
| NFKB1 | 7.00E-12 | PREDICTED: Capra hircus nuclear factor kappa B subunit 1 (NFKB1), transcript variant X1, mRNA | gcaatcgtgcagAAAgTAtTTGAACACTGGAAGCACGAATGACAGACGCCTGTgtAA |
| HP | 5.00E-42 | PREDICTED: Capra hircus haptoglobin (LOC102176354), mRNA | CACGTGGACAGACGCAGAGGACATAGGTGGGTCGCTGGATGCCAAAGGCAGCTTCCCCTGGCAGGCCAAGATGGTCTCCCACCATAACCTCATCTCGGGAGCCACACTA |
| PDK4 | 1.00E-14 | PREDICTED: Capra hircus pyruvate dehydrogenase kinase 4 (PDK4), mRNA | CCTAGAGGAACCCAAGCCTCATTGGAAGCATTGATCCAAACTGTGATGTGGCGGC |
| CPT1A | 1.00E-21 | PREDICTED: Capra hircus carnitine palmitoyltransferase 1A (CPT1A), mRNA | GGGTCCAGCGAGTCCCTCCAGTTGGCTCATCGTGGTTGTGGGCGTGATGTCGACCATGTACGCGCAAG |
| SUMO1 | 4.00E-15 | PREDICTED: Capra hircus small ubiquitin-like modifier 1 (SUMO1), transcript variant X1, mRNA | ACAGTGGGGGAGAAGGAGGAGAATATATTAAACTCAAAGTCATTGGACAGGATAGCAGTGANAA |
| APOE | 5.00E-19 | PREDICTED: Capra hircus apolipoprotein E (APOE), transcript variant X3, mRNA | CGACTCTCTGACAGGTGCAGGAGGAGCTGCTCAACACCCAGGTCATTCAGGAACTGACGGTAGGTC |
