# Supplementary figures and images for: 2,4-Thiazolidinedione in Well-Fed Lactating Dairy Goats: II. Response to Intra-Mammary Infection
Source: Vet Sci. 2019 Jun 5;6(2):52. doi: 10.3390/vetsci6020052 (PMC6632143; doi:10.3390/vetsci6020052)

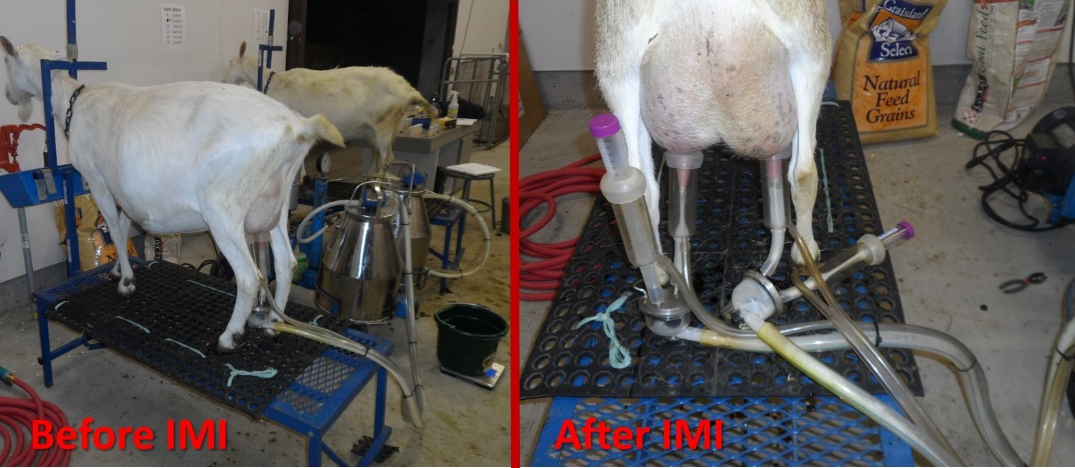

Supplement: Supplementary file 1 [file vetsci-06-00052-s001.zip › Figure S1.tif]

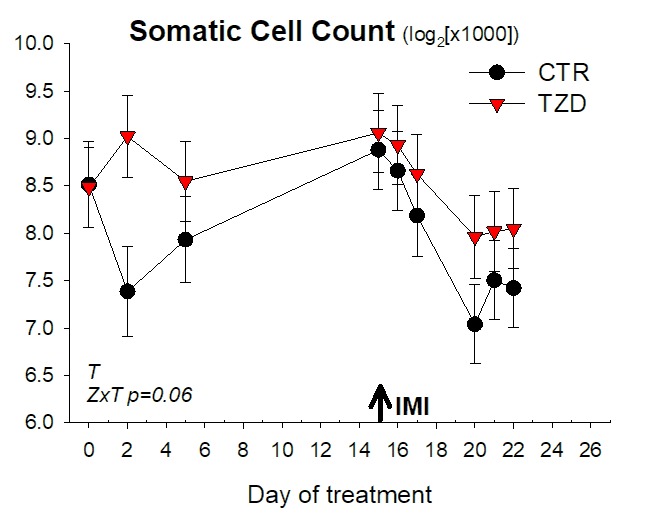

Supplement: Supplementary file 1 [file vetsci-06-00052-s001.zip › Figure S2.TIF]

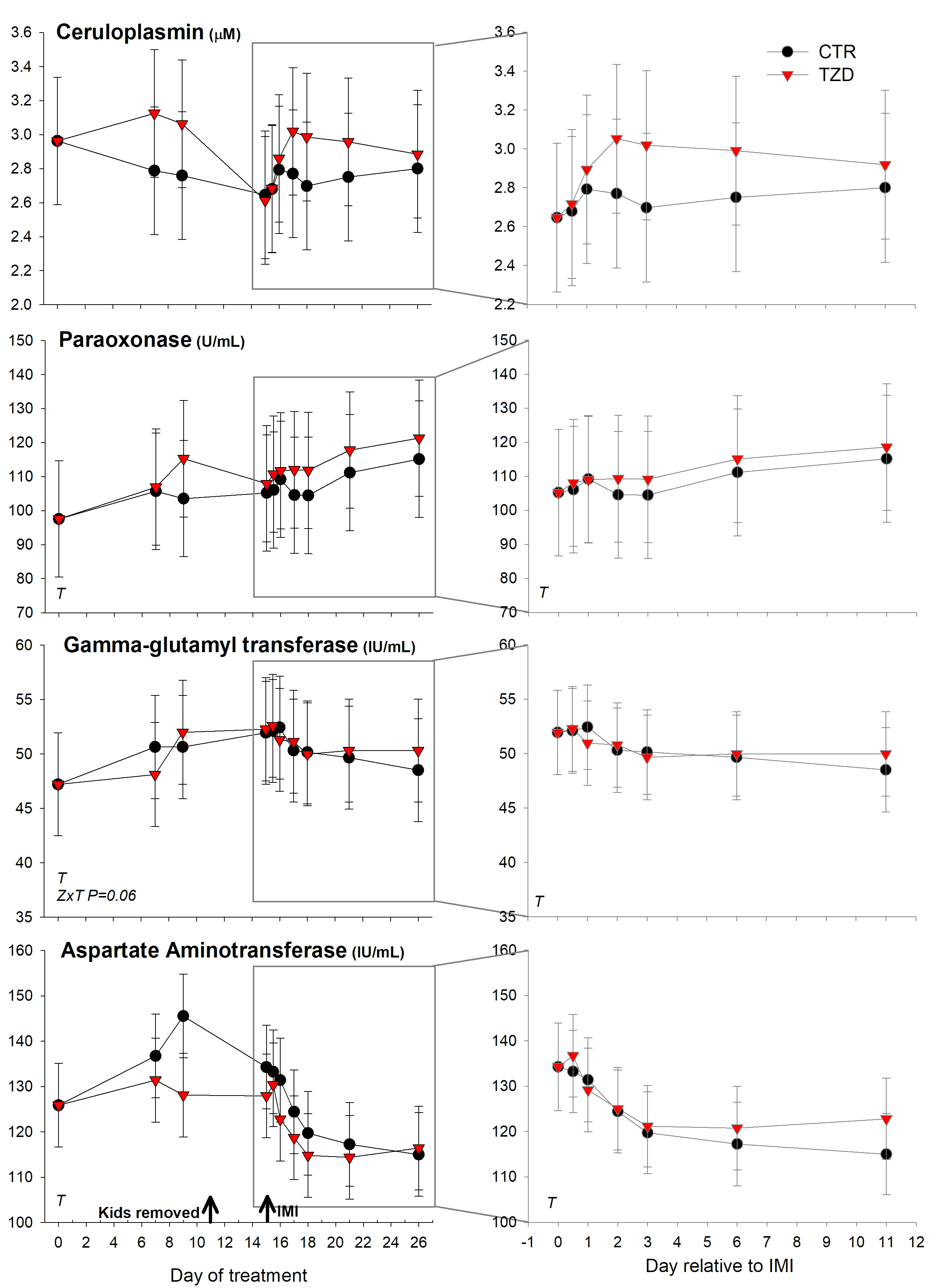

Supplement: Supplementary file 1 [file vetsci-06-00052-s001.zip › Figure S3.TIF]

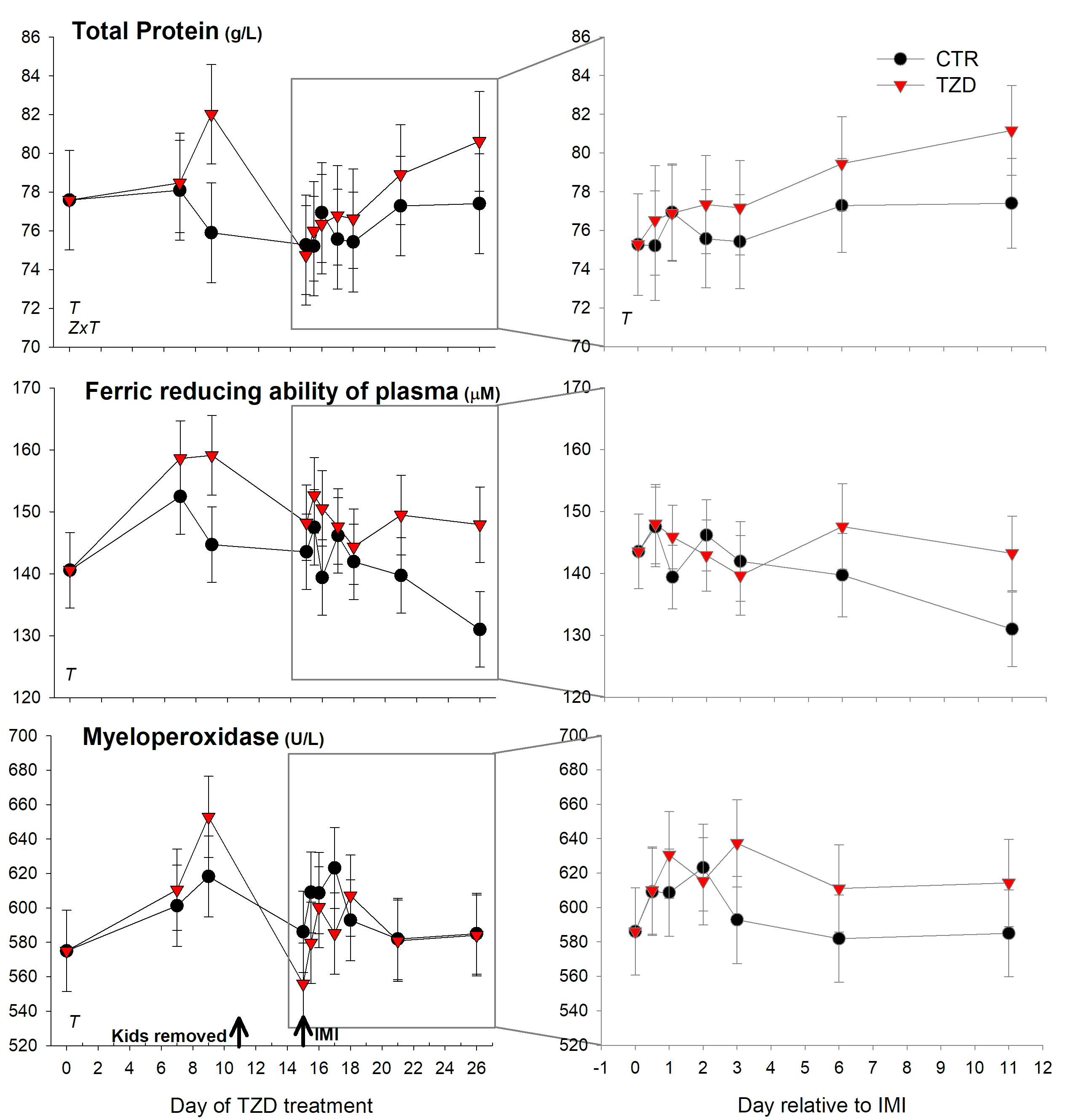

Supplement: Supplementary file 1 [file vetsci-06-00052-s001.zip › Figure S4.TIF]

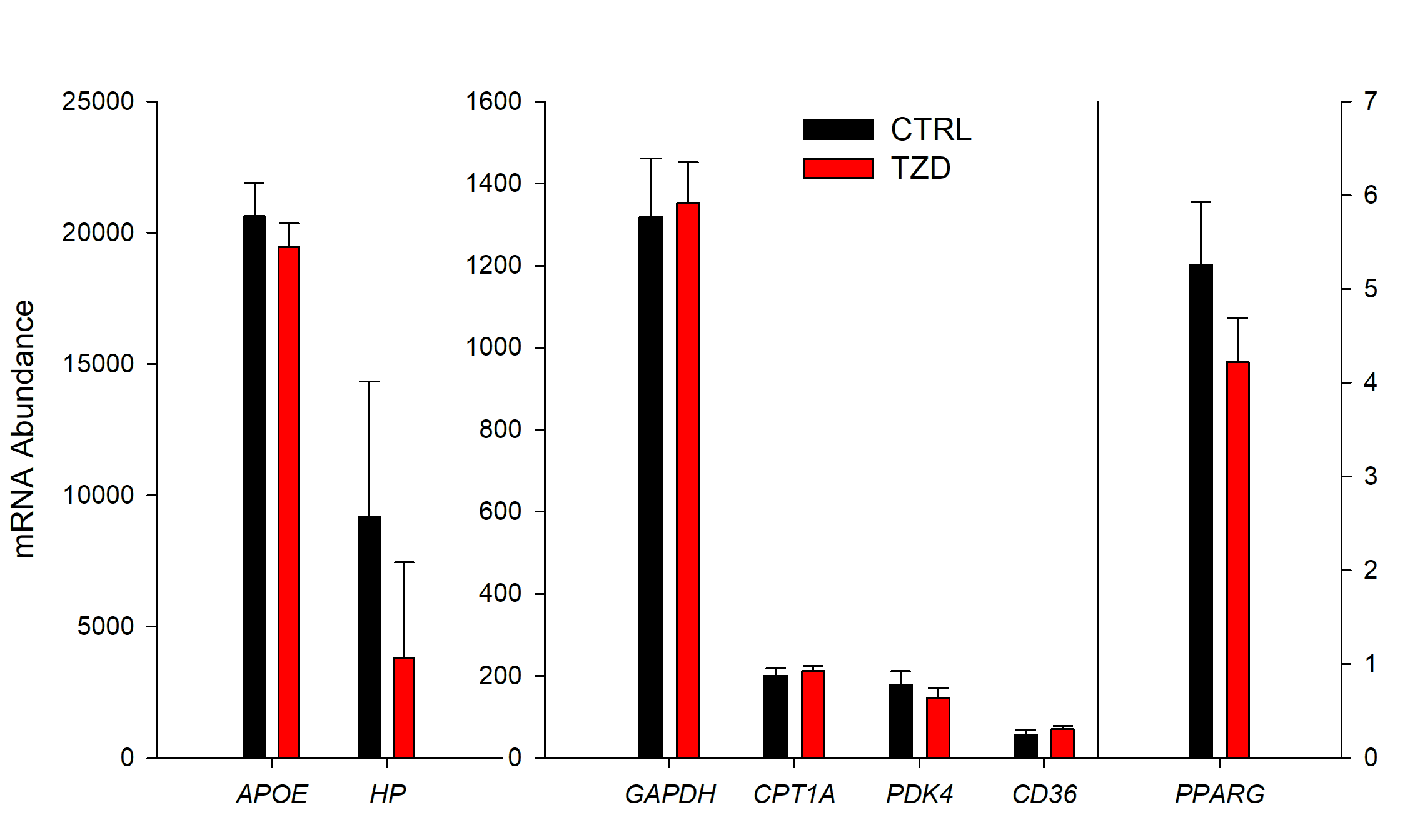

Supplement: Supplementary file 1 [file vetsci-06-00052-s001.zip › Figure S5.TIF]

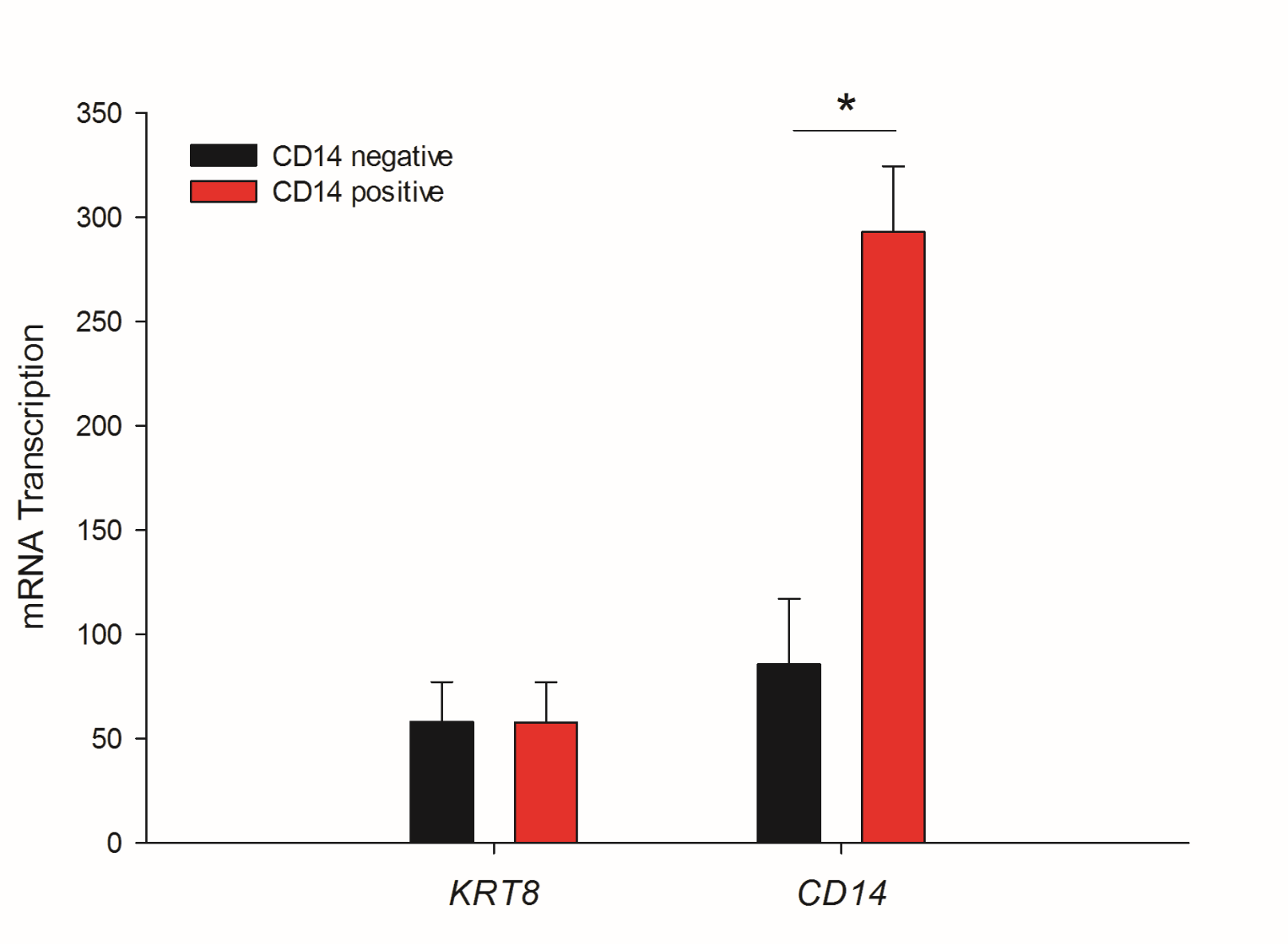

Supplement: Supplementary file 1 [file vetsci-06-00052-s001.zip › Figure S6.TIF]

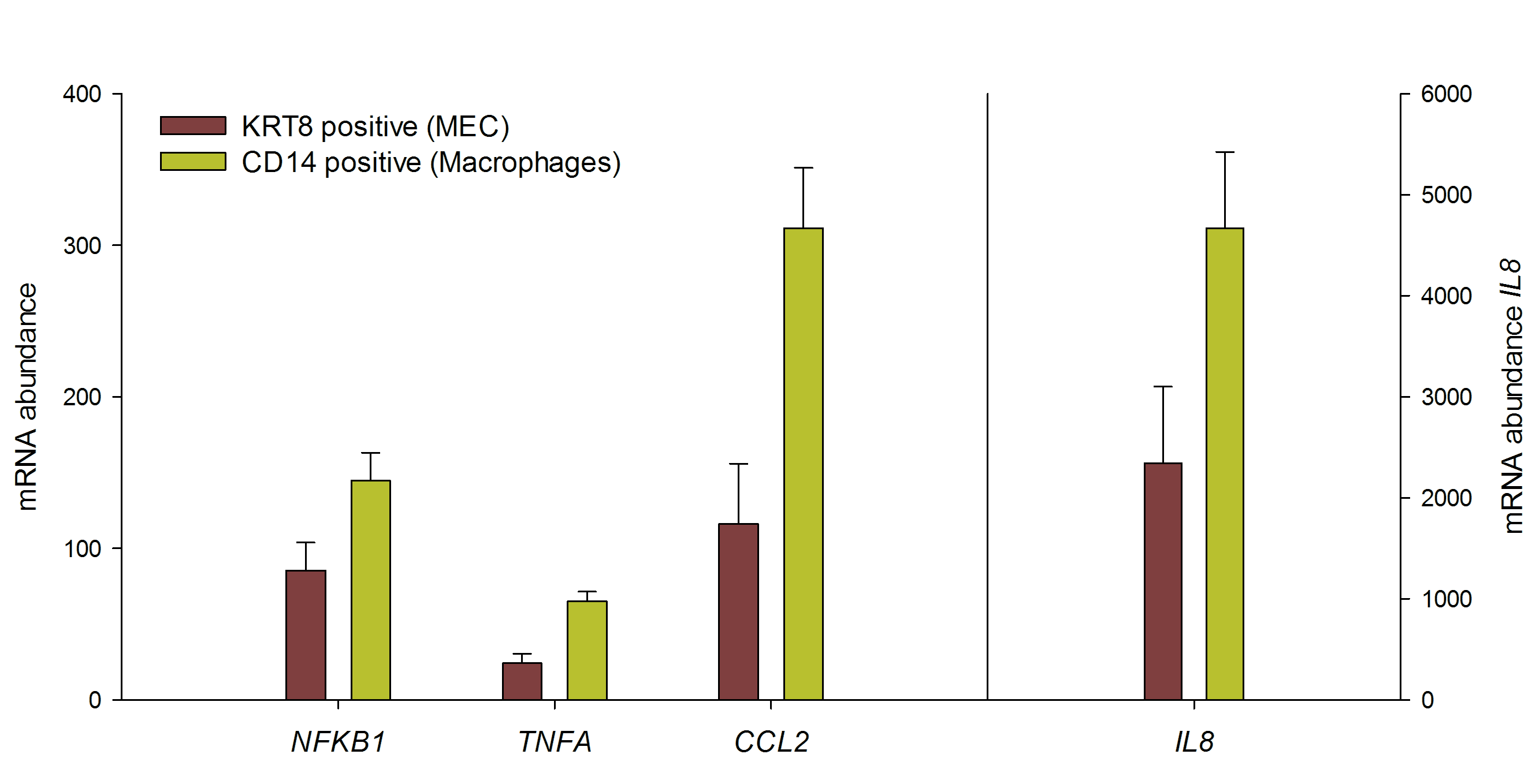

Supplement: Supplementary file 1 [file vetsci-06-00052-s001.zip › Figure S7.TIF]
